# Enhanced Strain Measurement Range of an FBG Sensor Embedded in Seven-Wire Steel Strands

**DOI:** 10.3390/s17071654

**Published:** 2017-07-18

**Authors:** Jae-Min Kim, Chul-Min Kim, Song-Yi Choi, Bang Yeon Lee

**Affiliations:** 1 Department of Marine and Civil Engineering, Chonnam National University, Yeosu 59626, Korea; jm4kim@jnu.ac.kr; 2 Department of Civil and Environmental Engineering, Graduate School, Chonnam National University, Yeosu 59626, Korea; kcm0555@nate.com; 3 INNOSE Tech., Incheon 21990, Korea; sychoi@innose.co.kr; 4 School of Architecture, Chonnam National University, Gwangju 61186, Korea

**Keywords:** FBG sensor, prestressed concrete, strand, strain, structural health monitoring

## Abstract

FBG sensors offer many advantages, such as a lack of sensitivity to electromagnetic waves, small size, high durability, and high sensitivity. However, their maximum strain measurement range is lower than the yield strain range (about 1.0%) of steel strands when embedded in steel strands. This study proposes a new FBG sensing technique in which an FBG sensor is recoated with polyimide and protected by a polyimide tube in an effort to enhance the maximum strain measurement range of FBG sensors embedded in strands. The validation test results showed that the proposed FBG sensing technique has a maximum strain measurement range of 1.73% on average, which is 1.73 times higher than the yield strain of the strands. It was confirmed that recoating the FBG sensor with polyimide and protecting the FBG sensor using a polyimide tube could effectively enhance the maximum strain measurement range of FBG sensors embedded in strands.

## 1. Introduction

The safety and serviceability of infrastructure are major design and construction considerations, and these factors as specified in standards such as design codes and specifications should be satisfied. However, structures deteriorate over time due to unexpected overload conditions or the degradation of structural members or materials, which can at times induce catastrophic failures of such structures. To address these issues, it is becoming more important to monitor the structural behavior of structures in real time using various types of sensors. Recently, many studies have attempted to realize technological advances in the area of structural health monitoring [1,2,3,4,5,6,7]. 

Optical fiber strain sensors including the interferometric in-fiber Fabry–Perot (F-P) sensors, distributed sensors utilizing optical time domain reflectometry (OTDR) or Brillouin scattering (BOTDA), and fiber Bragg grating (FBG), have widely employed for structural health monitoring (SHM) [1,2,3,4,6,7,8,9,10,11,12,13,14,15]. Especially, the FBG and F-P sensors are based on the phenomenon by which the refractive index of light of the sensor changes when the length of the sensor changes. An optical fiber sensor is not influenced by electromagnetic waves unlike sensors based on electrical resistance. Furthermore, optical fiber sensors have several advantages over conventional sensors, such as relatively small size, high durability, high sensitivity, and measurements at remote locations as far as several tens of kilometers. Particularly, the FBG sensor, a type of optical fiber sensor, is widely used because various physical quantities at several locations can be measured using only a single line of FBG sensors [8,9,10,11,12,13,14,15]. 

Previous studies reported that it is possible to measure the prestressing force of prestressed concrete (PSC) structures by attaching an FBG sensor to the surface of one of the strands used in such structures [16,17,18,19,20]. FBG sensors can be attached directly to such strands or in combination with a material similar to that of the strands. Kim et al. [21] introduced the new sensing technique of embedding an FBG sensor into the hollow king wire of a tendon, enabling the king wire to be used as a sensor as well as a prestressing wire. Kim et al. [2,22,23] reported that the prestressing force and strain of seven-wire steel strands, which are widely used for PSC members, can also be measured. 

**Figure 1:**
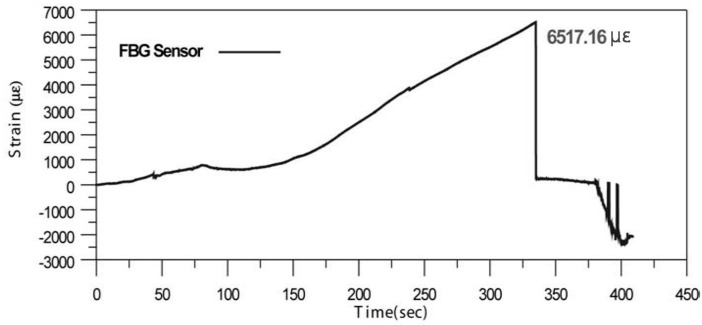
Strain measured using an FBG sensor embedded in the seven-wire steel strands [21].

Although previous studies established the feasibility of a sensing technique embedding FBG sensors in the seven-wire steel strands, it was observed that the maximum measurable strain of an FBG sensor embedded in the seven-wire steel strands was approximately 0.65% (6517.16 με) (Figure 1). The design strength of steel strands for prestressing concrete in a PSC bridge is 90% of the yield strength of the steel strands, and the strain corresponding to the yield strength is approximately 1%. Therefore, the design strain is approximately 0.9%, which requires the maximum measurement strain of sensors used to measure the strain or stress of steel strands to be higher than 1%.

The purpose of this study is to improve the maximum measurement range by exceeding the strain of 1.0% of an FBG sensor embedded in seven-wire steel strands. To achieve the purpose of the study, the reason for the lower maximum strain measurement range of an FBG sensor embedded in seven-wire steel strands relative to that of the FBG sensor itself is investigated and a new technique is proposed. In addition, the validity of the technique is assessed.

## 2. Seven-Wire Steel Strand with an Embedded FBG Sensor

### 2.1. FBG Sensor

An FBG sensor is a type of distributed Bragg reflector which is positioned in a short segment of optical fiber. It reflects a particular wavelength and transmits at another wavelength by creating a periodic variation in the refractive index of the fiber core by irradiation with an ultraviolet laser. The change in the physical properties of the FBG sensor due to an external load or a temperature change induces a change of the reflected wavelength. Therefore, the physical quantity can be measured from the relationship between the physical properties and the reflected wavelength of the FBG sensor.

**Figure 2:**
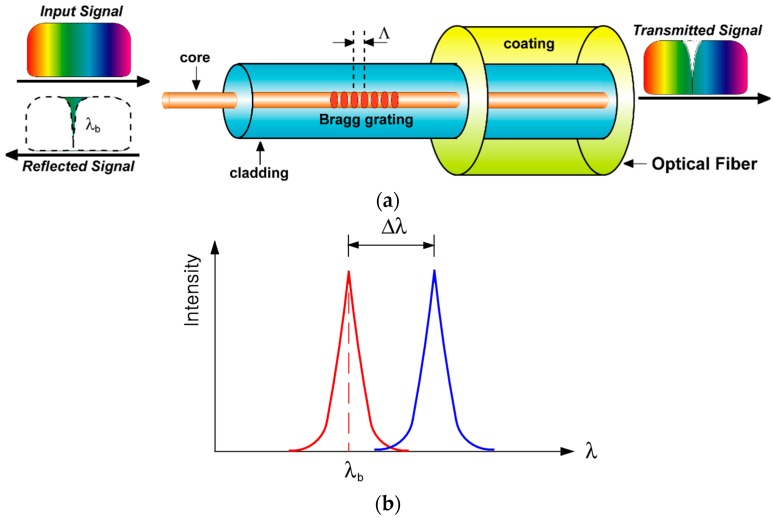
Schematic diagram of an FBG sensor: (**a**) principle of the FBG sensor; (**b**) wavelength shift due to deformation by a temperature change or external force [22].

Figure 2 shows a schematic diagram of the principle of an FBG sensor. Bragg gratings reflect light at a particular wavelength corresponding to these gratings, while light with other wavelengths is transmitted through the Bragg gratings. The reflected wavelength can be calculated by Equation (1).

(1) $$\lambda_{B}=2n_{eff}\Lambda ,$$

where $n_{eff}$ and Λ are the effective refractive index of the grating of the optical fiber and the grating period, respectively [24]. An external load or temperature change influences the spacing of the grating, which induces a change in the reflected wavelength. The change in the reflected wavelength (Δλ) can be calculated with Equation (2).

(2) $$\frac{\Delta \lambda }{\lambda_{B}}=\left(1-P_{e}\right)\Delta \varepsilon +\left(\alpha +\xi \right)\Delta T,$$

In this equation, $P_{e}$ is the effective strain-optic constant, α is the thermal expansion coefficient, Δε is the effective strain, ΔT is the temperature change, and ξ is the thermo-optic coefficient, which represents the relationship between the temperature change and the change in the refractive index [15].

FBG sensors offer many advantages, as described in the previous section. However, FBG sensors are also brittle because they are composed of a glass-based material, implying that they are easily damaged when installed. Therefore, some form of protection is necessary. Iten [25] investigated protection methods for optical fiber-based sensors and compared the protection performance of each method. Test results showed that two layers of protection composed of soft plastic as an infill between the optical fiber and an external tube and hard plastic as an external tube can effectively improve the maximum strain measurement range of optical fiber.

### 2.2. Structure of the Seven-Wire Steel Strand with an Embedded FBG Sensor

**Figure 3:**
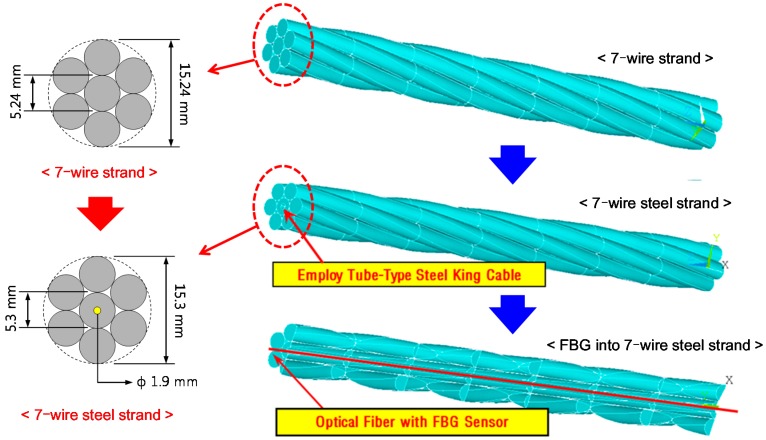
Concept of a seven-wire steel strand embedding an FBG sensor.

Seven-wire steel strands, which are widely used to impart prestress to concrete of PSC bridges, are composed of one straight king wire and six helical wires. Figure 3 shows schematic diagrams of a general seven-wire strand and a seven-wire strand with an embedded FBG sensor. The king wire is replaced with a hollow steel tube and an FBG sensor is installed inside the tube. An FBG sensor can be fixed with an epoxy adhesive for integration with the steel tube [2,9,21,22,23]. Coefficients of the FBG sensor given in reference [23], which were identified for the sensor itself and the seven-wire strand with the sensor by the calibration tests for strain and temperature, were employed in this study, as follows: *P_e_* = 0.22, α = 1.20 × 10^−5^/°C, and ξ = 5.67 × 10^−6^/°C.

## 3. Limitations and Performance Enhancements of FBG Sensors Embedded in Seven-Wire Steel Strands

### 3.1. Effect of the Coating Type on the Maximum Strain Measurement Range of Optical Fiber

**Figure 4:**
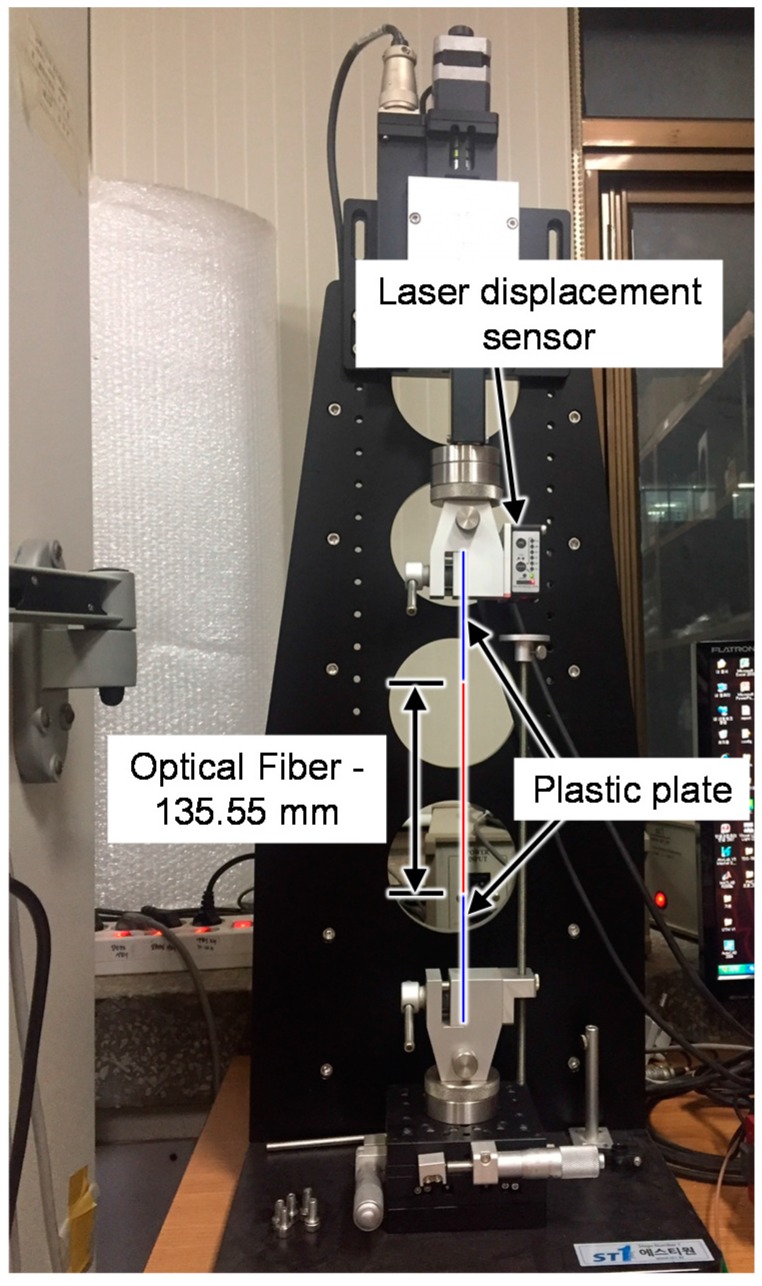
Tension test setup for the optical fiber.

**Figure 5:**
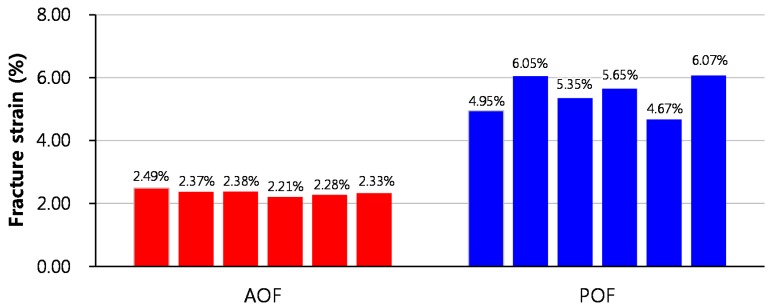
Fracture strains of optical fiber samples.

An FBG sensor is generally manufactured in three steps: removal of the acrylate coating of the optical fiber, writing of periodic variations of the refractive index, and recoating of the surface of the optical fiber with acrylate. Previous studies proposed a new recoating method for optical fiber with polyimide during the manufacturing process and found that the damage resistance and durability of the optical fiber were improved [26,27,28]. In order to investigate the effect of recoating materials on the maximum strain measurement range of optical fiber, two types of optical fibers were manufactured using acrylate and polyimide as recoating materials and the fracture strain was measured. Acrylate with a tensile strength of 10.7 MPa, an elastic modulus of 47.4 MPa, and a density of 0.94 g/cm^3^; and polyimide with a tensile strength of 102 MPa, an elastic modulus of 4.28 GPa, and a density of 1.48 g/cm^3^ were used in this study. Figure 4 shows the test setup used to measure the fracture strain of the optical fiber. A tensile load was applied under displacement control at a speed of 0.6 mm/min. Six specimens were measured for each optical fiber. Figure 5 shows the fracture strains of each specimen. The fracture strain of the POF specimens was 2.3 times higher than that of the AOF specimens. From the test results, it was confirmed that the maximum strain measurement range of optical fiber can be improved by the recoating of the optical fiber with polyimide.

### 3.2. Effect of the Coating Type on the Maximum Strain Measurement Range of an Optical Fiber Embedded in a Strand

**Figure 6:**
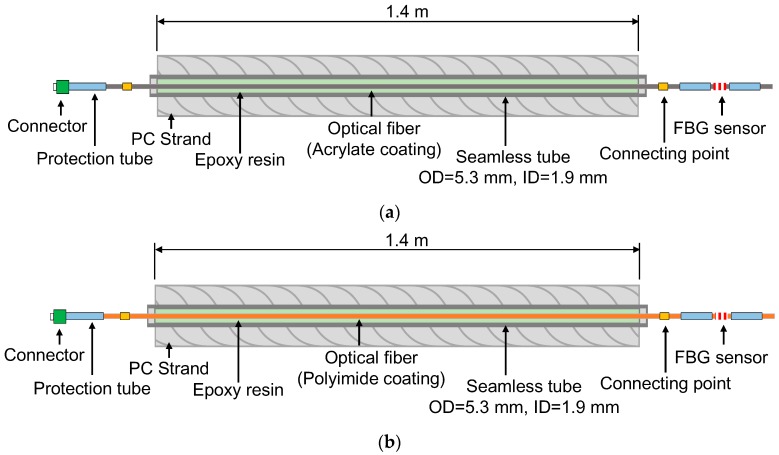
Schematic diagram of strand-embedded optical fibers recoated with two types of materials: (**a**) OF-A (recoated with acrylate); (**b**) OF-P (recoated with polyimide).

In order to investigate the maximum strain measurement range of optical fibers embedded in strands according to type of recoating material used, two types of smart strand embedding optical fibers recoated with acrylate and polyimide were manufactured and tension tests were performed. The diameter of the optical fiber in each case was 0.25 mm, and the outer and inner diameters of the steel tube were 5.3 mm and 1.9 mm, respectively. The steel tube is a seamless tube made of stainless steel. Figure 6 shows a schematic diagram of the seven-wire strand with embedded optical fibers as used in the tension test. For the OF-A and OF-P specimens, the optical fibers were recoated with acrylate and polyamide, respectively. Epoxy resin was injected for bonding between the optical fiber and the seamless tube. FBG sensors were installed at one end of each specimen to measure the fracture strain of the optical fibers embedded in the strands. 

**Figure 7:**
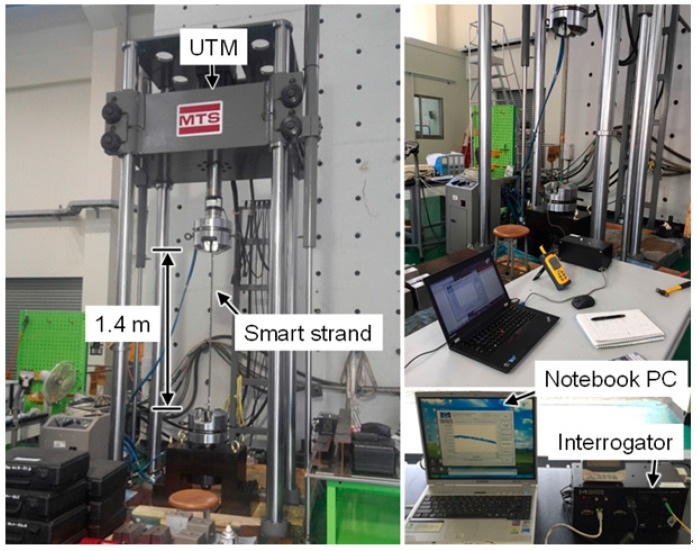
Tension test setup of strands using a universal testing machine.

**Figure 8:**
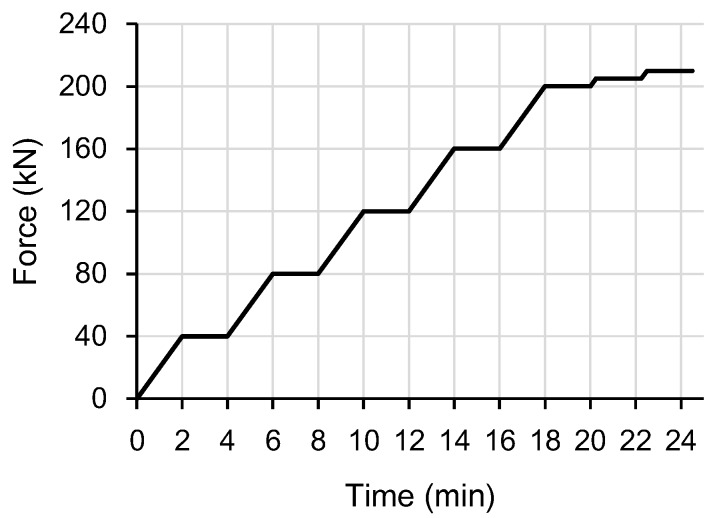
Load profile of the tension test.

**Figure 9:**
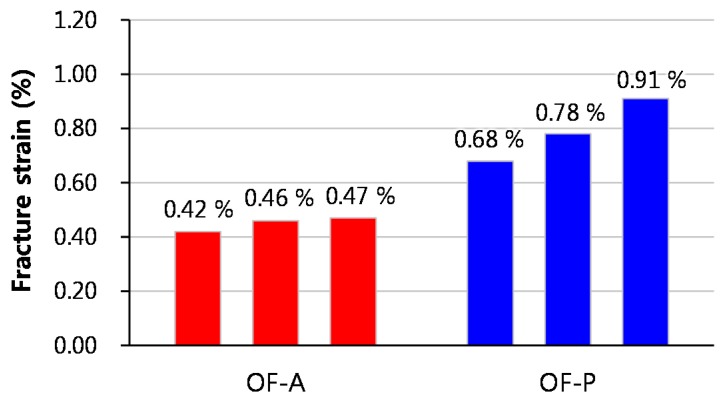
Fracture strains of optical fibers embedded in a strand according to the recoating material used.

Figure 7 shows the tension test setup of the strands with a length of 1.4 m. The tensile load was applied using a universal testing machine under load control with the load profile shown in Figure 8. The loading speed was 20.0 kN/min up to 210 kN. Figure 9 shows the fracture strains, which are identical to the maximum strain measurement range, of each specimen. The OF-P specimen showed greater fracture strain by 76% than the OF-A specimen. From these test results, the maximum strain measurement in a strand using an optical fiber-based sensor can be improved by recoating the optical fiber with polyimide. However, the fracture strains of the OF-P and OF-A specimens were 81% and 86% lower than those of the AOF and POF specimens, respectively. This indicates that the fracture strain of an optical fiber decreases dramatically when it is embedded in a strand.

### 3.3. Enhancement Method of the Maximum Strain Measurement Range

**Figure 10:**
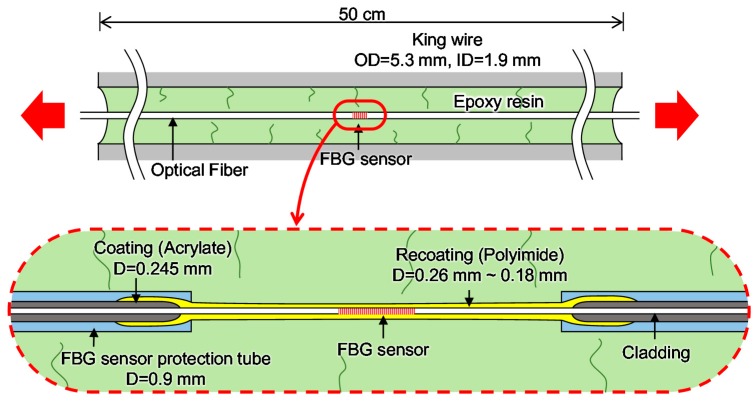
Schematic diagram of the king wire of the smart strand embedding the FBG sensor subject to a tensile load.

Figure 10 shows a schematic diagram of the king wire with the FBG sensor subject to a tensile load. Epoxy resin is used to integrate the FBG sensor and the king wire tube in the strand. It was assumed that cracks occur in the epoxy resin, which consequently induces local damage in the optical fiber. This is the main reason for the decrease of the maximum strain measurement range of the optical fiber-based sensor embedded in a strand. 

**Figure 11:**
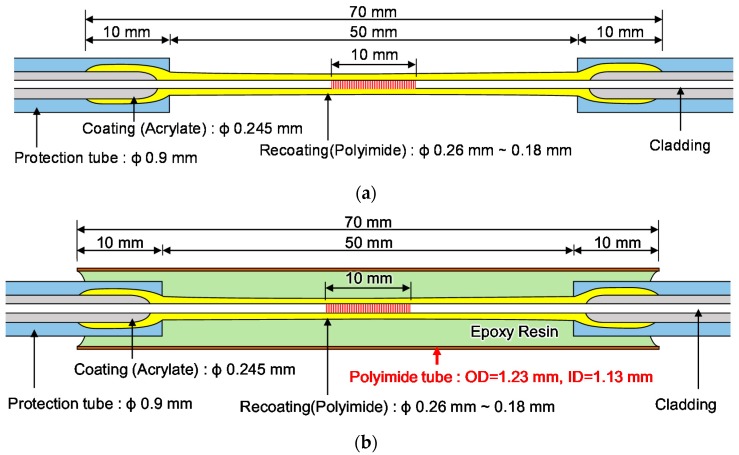
Schematic diagram of FBG sensors: (**a**) FBG-P-NP (without a polyimide tube); (**b**) FBG-P-PP (with a polyimide tube).

Figure 11a shows a schematic diagram of a normal FBG sensor. This study suggests a new method by which to improve the maximum strain measurement range of an FBG sensor embedded in a strand. Figure 11b shows a schematic diagram of the proposed method to minimize local damage to an FBG sensor embedded in a strand. The FBG sensor is protected by the polyimide tube and epoxy resin is injected between the FBG sensor and the polyimide tube for the integration of the sensor and the tube. Two types of FBG sensors, i.e., a normal FBG sensor (FBG-P-NP) and a new FBG sensor (FBG-P-PP), for embedment in a strand were manufactured. The polyimide tube had an outer diameter of 1.23 mm and an inner diameter of 1.13 mm, and typical values of the tensile strength and elastic modulus of the polyimide tube given by the manufacturer at 23 °C were 231 MPa and 2.50 GPa, respectively. The epoxy resin with a viscosity of 150 cPs, a Shore D hardness of 85, and a lap shear strength of 14 MPa was used to integrate the FBG sensor and polyimide tube and also the polyimide tube and the king wire tube.

### 3.4. Validation of the Proposed Method

**Figure 12:**
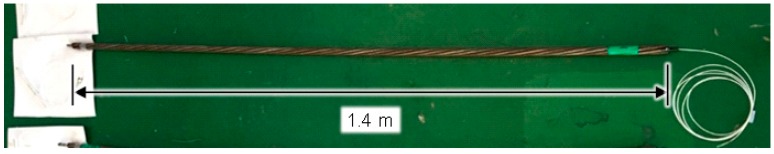
Specimen used in the tension test of a smart strand embedding the FBG sensor as proposed in this study.

To assess the validity of the proposed technique, two types of strands with the length of 1.4 m were manufactured. The first is a normal strand embedding a normal FBG sensor (FBG-P-NP), and the second is the new strand embedding a new FBG sensor (FBG-P-PP). Seven specimens were prepared to check the validity of the test results. Figure 12 shows specimen used in the tension test of the new strand embedding the FBG sensor proposed in this study. The tensile load was applied under load control by a universal testing machine, identical to the process described in Section 3.2.

**Figure 13:**
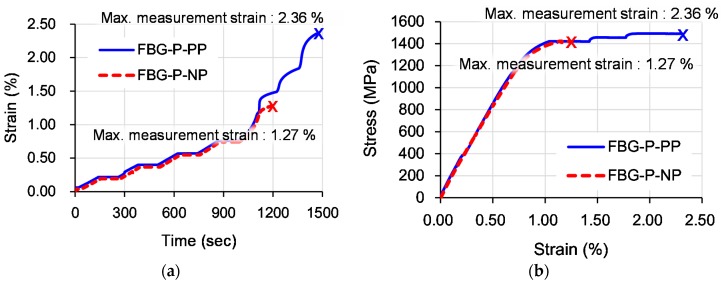
Representative test results: (**a**) tensile strain and time curves; (**b**) tensile stress and strain curves.

Figure 13 shows the representative tensile strain and time curves and the tensile stress and strain curves of FBG-P-NP and FBG-P-PP specimens. It can be seen that the maximum strain measurement range of the FBG-P-PP specimen was higher than that of the FBG-P-NP specimen. 

**Figure 14:**
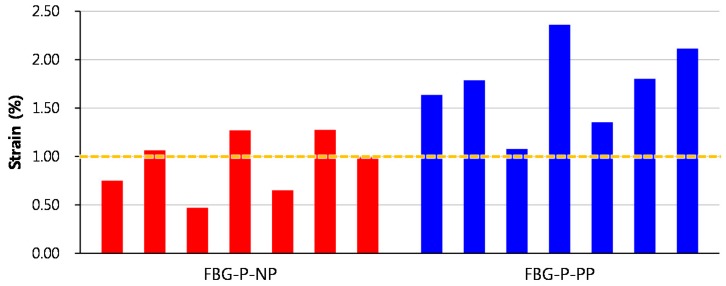
Maximum strain measurement ranges of the FBG sensors.

**Figure 15:**
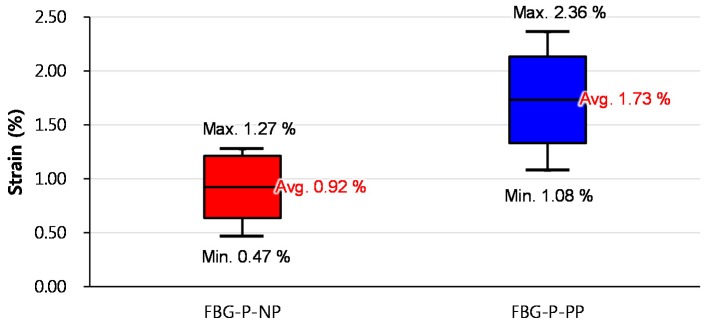
Comparison of maximum strain measurement ranges of FBG sensors.

Figure 14 shows the maximum strain measurement range of all specimens and Figure 15 shows a box-and-whisker plot of the maximum strain measurement ranges of the FBG-P-NP and FBG-P-PP specimens. The average maximum strain measurement range of FBG-P-NP was 0.92%, which is lower than that of the yield strain (approximately 1%) of the steel strand. In contrast, the average maximum strain measurement range of FBG-P-PP was 1.73%, which is 88% higher than that of FBG-P-NP. Furthermore, all specimens showed a higher maximum strain measurement range than the yield strain of the steel strand. From these test results, it was confirmed that the protection method of the FBG sensor using the polyimide tube can effectively increase the maximum strain measurement range of an FBG sensor embedded in a strand. It was also validated that cracks in the epoxy resin can damage the FBG sensor. Therefore, the proposed FBG sensing system can be applied to monitor the strain in strands, particularly when it exceeds the yield strain of the strands.

## 4. Conclusions

This study proposed a new FBG sensing technique to enhance the maximum strain measurement range of FBG sensors embedded in strands to measure the strain on the strands in PSC structures. A series of experiments were performed to investigate the effects of recoating materials and of tube used to protect the FBG sensor on the maximum strain measurement range of an FBG sensing system and to assess the validity of the newly proposed FBG sensing system. The following conclusions can be drawn from the current test results:

1. The fracture strain of an optical fiber recoated with acrylate was 0.45%. On the other hand, the fracture strain of an optical fiber recoated with polyimide was 0.79%, which is 75% higher than that of the optical fiber recoated with acrylate. From these test results, it was confirmed that the fracture strain of optical fibers can be increased by recoating the optical fibers with polyimide.
2. The maximum strain measurement range of a normal FBG sensor embedded in a strand was 0.92%. On the other hand, the maximum strain measurement range of the new FBG sensor, which was protected by the polyimide tube, embedded in a strand was 1.73%, which was 88% higher than that of the normal FBG sensor.
3. All steel strand specimens adopting the new FBG sensing system, in which the optical fiber was recoated with polyimide and the FBG sensor was protected by the polyimide tube, showed higher maximum strain measurement ranges which exceeded 1.0%, which is the yield strain of the steel strands used, with measurement possible up to a strain level of 2.36%. Therefore, the new FBG sensing technique can be effectively applied to monitor the strain on strands in PSC structures.
